# Efficient and Selective Adsorption of Cationic Dye Malachite Green by Kiwi-Peel-Based Biosorbents

**DOI:** 10.3390/molecules28145310

**Published:** 2023-07-10

**Authors:** Yanjun Zhao, Xintong Liu, Wenhui Li, Suyun Pei, Yifan Ren, Xinyang Li, Chen Qu, Chuandong Wu, Jiemin Liu

**Affiliations:** 1 School of Chemistry and Biological Engineering, University of Science and Technology Beijing, No. 30 Xueyuan Road, Haidian District, Beijing 100083, China; zhaoyj@ustb.edu.cn (Y.Z.); liwh@ustb.edu.cn (W.L.); psy3179017442@163.com (S.P.); 18811503718@163.com (Y.R.); quchen@ustb.edu.cn (C.Q.); 2 School of Light Industry, Beijing Technology and Business University, No. 33 Fucheng Road, Haidian District, Beijing 100048, China; liuxt@btbu.edu.cn; 3 China Testing & Certification International Group Co., Ltd., No. 1 Guanzhuang Road, Chaoyang District, Beijing 100024, China; lxyustb@163.com; 4 Beijing Institute of Graphic Communication, No. 1 Xinghua Street (Section 2), Daxing District, Beijing 102600, China

**Keywords:** kiwi peel, acid-modified adsorbent, selective adsorption, malachite green, nitric acid modification

## Abstract

In this study, pristine kiwi peel (KP) and nitric acid modified kiwi peel (NA-KP) based adsorbents were prepared and evaluated for selective removal of cationic dye. The morphology and chemical structure of KP and NA-KP were fully characterized and compared, and results showed nitric acid modification introduced more functional groups. Moreover, the adsorption kinetics and isotherms of malachite green (MG) by KP and NA-KP were investigated and discussed. The results showed that the adsorption process of MG onto KP followed a pseudo-second-order kinetic model and the Langmuir isotherm model, while the adsorption process of MG onto NA-KP followed a pseudo-first-order kinetic model and the Freundlich isotherm model. Notably, the Langmuir maximum adsorption capacity of NA-KP was 580.61 mg g^−1^, which was superior to that of KP (297.15 mg g^−1^). Furthermore, thermodynamic studies demonstrated the feasible, spontaneous, and endothermic nature of the adsorption process of MG by NA-KP. Importantly, NA-KP showed superior selectivity to KP towards cationic dye MG against anionic dye methyl orange (MO). When the molar ratio of MG/MO was 1:1, the separation factor (α_MG/MO_) of NA-KP was 698.10, which was 5.93 times of KP. In addition, hydrogen bonding, π-π interactions, and electrostatic interaction played important roles during the MG adsorption process by NA-KP. This work provided a low-cost, eco-friendly, and efficient option for the selective removal of cationic dye from dyeing wastewater.

## 1. Introduction

With the growing development of industries, the consequent environmental problems have drawn increasing concerns. Numerous kinds of organic dyes have been widely employed in various industries [1,2], such as textile, cosmetics, leather, foods, pharmaceutical, etc. It is reported that approximately 10–15% of the consumed dyes are discharged into the hydrosphere during processing, which poses a serious threat to water resources, the aquatic environment, and human health [3]. According to the functionality of the chromophore group, organic dyes can be classified into cationic, anionic, and nonionic dyes [4,5]. Cationic dyes are considered to have high toxicity and carcinogenicity, which can cause harmful effects on human health even at extremely low levels [6]. For example, malachite green (MG) is a typical cationic dye, which was reported to be carcinogenic, teratogenic, and genotoxic, and can pose a threat to animals and human beings even at even at extremely low levels [7,8]. Therefore, the development of efficient technologies for selective cationic dye removal is crucial, and significant efforts have been made to develop various treatment technologies [9,10], such as membrane filtration, ultrafiltration, adsorption, and advanced oxidation processes, etc. Among these methods, adsorption presented the advantages of simple operation, high removal efficiency, and cost-effectiveness [9,11]. Even so, the exploration of sustainable, low-cost, highly efficient, and selective adsorbents remains as the fundamental issue in adsorptive removal of toxic dyes [7].

Due to the characteristics of nontoxic, biodegradable, and extensive resources, agricultural by-product is considered as a promising alternative for developing efficient adsorbents [12,13]. Recently, various agricultural by-products have been evaluated as raw materials to develop dye adsorbent, such as garlic peels [14], banana peels [15], potato peels [16], orange peels [17], cactus fruit peels [18], sugarcane bagasse [19], *Artocarpus odoratissimus* leaves [20], *Gigantochloa* bamboo [21], and so on. Kiwi (*Actinidia deliciosa*) is a popular and widely cultivated fruit, which is rich in vitamin, mineral, and dietary fiber. In addition to being directly consumed, kiwi fruit could be processed into other foods or drinks, such as fruit jam, canned fruit, jelly, fruit juice, or fruit wine etc., which leads to a large amount of kiwi peel being discarded. The development of a kiwi-peel-based biosorbent is not only a promising way to remove pollutants, but also an efficient solution for high-value-added utilization of kiwi peel waste [22,23,24]. Nevertheless, literature about kiwi-peel-based adsorbents was limited when compared with other fruit peels, especially with regard to their application in dye removal [25,26,27]. Moreover, research on developing a kiwi-peel-based adsorbent for selective removal of cationic dyes remained absent until now, and this topic requires further investigation.

It is worth noting that most pristine agricultural by-products suffer from low adsorption capacity and selectivity, which restricts their practical application in wastewater treatment. It is well acknowledged that chemical modification could introduce functional groups and active binding sites on the surface of agricultural by-products, thus a variety of chemical regents have been applied to improve adsorption capacity and selectivity, such as acids, bases, oxidants, inorganic salts, and organic reagents [9,10,28,29,30,31]. However, previous studies on kiwi-peel-based adsorbents for dye removal only involved modification of carbonization [25], polyacrylamide-grafting [26], and CTAB/H_2_O_2_ [27]. Hence, the exploration of other modification methods and the investigation of the adsorption mechanism of kiwi-peel-based biosorbents for dye removal were crucial.

In this regard, we prepared pristine kiwi peel (KP) and nitric acid modified kiwi peel (NA-KP) based biosorbent and intensively investigated and compared their adsorption capacity and selectivity for cationic dye. In order to investigate the influence of nitric acid, the physical and chemical properties of KP and NA-KP were characterized and compared. Furthermore, the adsorption kinetics, isotherms, and thermodynamics of MG adsorption onto KP and NA-KP were fully studied and analyzed. Specifically, in order to evaluate the selectivity of KP and NA-KP towards cationic dye, malachite green (MG) and methyl orange (MO) were selected as model cationic and anionic dyes, and the separation factors of KP and NA-KP were calculated in the binary mixtures. Furthermore, the possible adsorption mechanism of MG onto NA-KP was discussed and proposed. This work not only provided a promising solution for the utilization of wasted kiwi peels, but also developed an efficient, selective, low-cost, and eco-friendly biosorbent for cationic dye removal.

## 2. Results and Discussion

### 2.1. Characterization of Kiwi-Peel-Based Adsorbents

**Figure 1:**
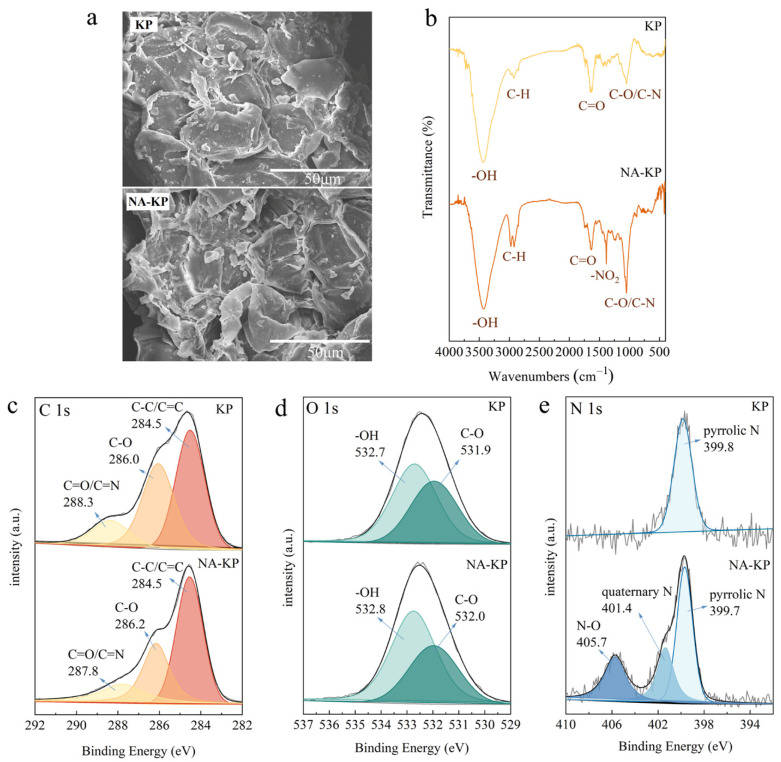
Characterization of KP and NA-KP: SEM morphologies (**a**) FT-IR spectra (**b**), XPS spectra: C 1s (**c**), O 1s (**d**), and N 1s (**e**).

The surface morphology of KP and NA-KP are shown in Figure 1a. Both KP and NA-KP featured reticulated convex structure, while the structure of NA-KP was more disordered due to the corrosion by nitric acid. However, the change in morphology after nitric acid modification was not obvious, which might be due to the moderate activation process by the nitric acid.

Furthermore, the surface functional groups of KP and NA-KP were investigated by the FT-IR spectra of KP and NA-KP from 4000 to 400 cm^−1^. As observed in Figure 1b, the intense and broad peaks in the range of 3792–3054 cm^−1^ are attributed to the stretching vibration of -OH, the peaks observed in the range of 2977–2921 cm^−1^ are the vibration of aromatic C-H, the peaks observed at 1637 cm^−1^ and 1632 cm^−1^ are assigned to C=O stretching vibrations, and the peaks at 1047 cm^−1^ and 1050 cm^−1^ are attributed to C-O or C-N stretching vibrations [14]. It can be seen from the FT-IR spectra that there are differences between KP and NA-KP. First of all, the new peak observed in the spectra of NA-KP at 1385 cm^−1^ is attributed to -NO_2_ stretching vibrations [32], which indicated that modification by nitric acid might cause an increase in the -NO_2_ functional group. Moreover, the peak at 1050 cm^−1^ of C-O/C-N in NA-KP became relatively stronger, which also indicated the increase in functional groups after HNO_3_ modification.

Moreover, the major components of KP and NA-KP were determined by elemental analysis (EA) and the results are shown in Table S1. Results showed that the contents of C, O, and H were not obviously changed after HNO_3_ modification, while the content of N significantly increased from 1.02% to 2.70%, which also indicated the increase of functional groups containing N.

The X-ray photoelectron spectroscopy (XPS) of KP and NA-KP were determined to further investigate the chemical statement of main components. As shown in Figure 1c, the C 1s deconvolution spectra of KP and NA-KP both showed three peaks, which were attributed to C-C/C=C, C-O, and C=O/C=N, respectively [11]. According to the peak areas, the percentage of C-C/C=C, C-O, and C=O/C=N in KP were 45.08%, 40.25%, and 14.67%, while in NA-KP were 50.18%, 31.02%, and 18.80%, respectively. The decrease in C-O and the increase in C=O/C=N in NA-KP might be caused by the increase of functional groups containing N. In the deconvolution spectra of O 1s depicted in Figure 1d, both KP and NA-KP observed two peaks, which were attributed to C-O and -OH [33,34]. According to the peak areas, the percentage of C-O and -OH were 42.81% and 57.19% in KP, and were 37.47% and 62.53% in NA-KP, which indicated the increase of -OH after HNO_3_ modification. Specifically, the deconvolution spectra of N 1s shown in Figure 1e could reveal obvious differences between KP and NA-KP. For KP, there was only one peak attributed to pyrrolic N. However, the spectrum of NA-KP displayed three peaks, which could be assigned to N-O, quaternary N, and pyrrolic N [35]. The results of XPS analysis further confirmed the increase in functional groups after HNO_3_ modification, which might be favorable for enhancing adsorption capacities for cationic dyes. Overall, the results of the characterization of KP and NA-KP suggested that nitric acid could react with structures of low activity, changing them into active functional groups such as -OH, -COOH and -NO_2_, which might be beneficial for enhancing adsorption capacity and selectivity.

### 2.2. Adsorption Kinetics

**Figure 2:**
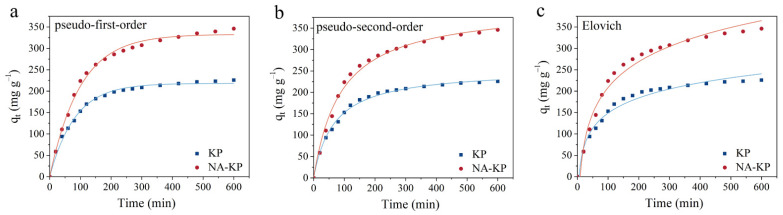
Kinetic model fitting of adsorption data by KP and NA-KP: pseudo-first-order model (**a**), pseudo-second-order model (**b**), and Elovich model (**c**).

As shown in Figure 2, the adsorption performances of MG by KP and NA-KP were evaluated under identical conditions, and the adsorption kinetics were analyzed. With the increase in contact time, the adsorption capacities of KP and NA-KP both increased rapidly at the beginning, then increased slowly until reaching the equilibrium trends. Importantly, the adsorption capacity of NA-KP was obviously superior to KP, which might be attributed to the increase in functional groups after HNO_3_ modification. Moreover, in order to further understand possible mechanism of MG adsorption onto KP and NA-KP, non-linear fitting of pseudo-first order, pseudo-second order and Elovich kinetic models were performed. The equations and corresponding parameters are presented in Table 1, and the meanings of kinetic models are shown in Text S1.

Generally, the adsorption data of KP fitted the pseudo-second-order kinetic model best with a correlation coefficient (R^2^) of 0.9958, while the adsorption data of NA-KP fitted the pseudo-first-order kinetic model best with an R^2^ of 0.9943. This result indicated that the dominant rate-controlling factors of the adsorption process by KP might be chemisorption mechanisms, whereas adsorbate diffusion might be a dominant controlling step for adsorption process by NA-KP [36,37]. Furthermore, the Elovich model fitted better with the kinetic data of NA-KP (R^2^ = 0.9822) than KP (R^2^ = 0.9791), which indicated that the adsorption of MG by NA-KP might be a heterogeneous physicochemical adsorption. Based on the calculated equilibrium adsorption capacities (q_e,cal_) obtained by pseudo-first-order and pseudo-second-order models, the adsorption capacity of NA-KP was about 1.53–1.57 times that of KP under identical parameters. Moreover, the initial adsorption rate h of MG onto NA-KP (4.48 mg g^−1^ min^−1^) was higher than that of KP (3.80 mg g^−1^ min^−1^). This might be due to the increase in functional groups after HNO_3_ modification, which would have led to an increase of surface active sites favorable for MG adsorption.

### 2.3. Adsorption Isotherms

**Figure 3:**
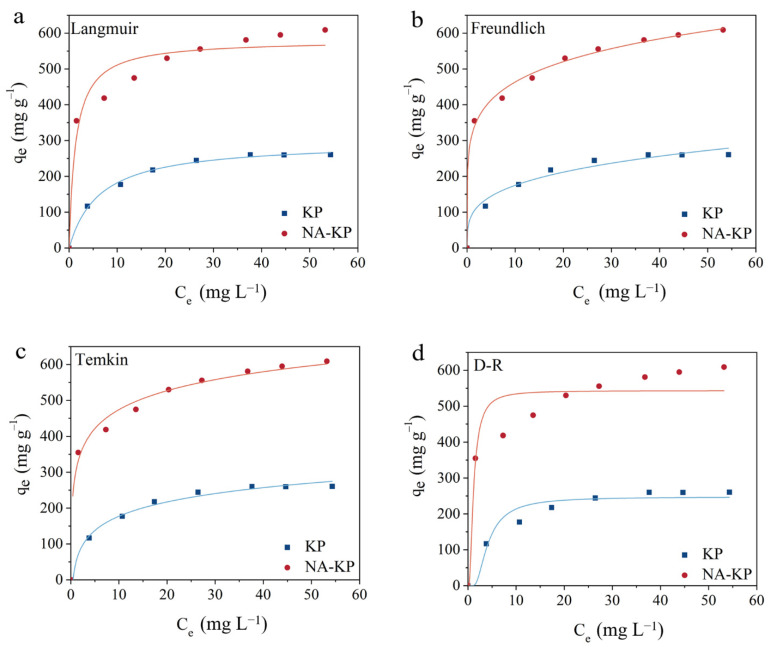
Isotherm model fitting of equilibrium data by KP and NA-KP: Langmuir (**a**), Freundlich (**b**), Temkin (**c**), and Dubinin-Radushkevich (**d**).

The adsorption isotherms were evaluated to further investigate the adsorption behaviors of MG onto KP and NA-KP. As shown in Figure 3, Langmuir, Freundlich, Temkin, and Dubinin-Radushkevich (D-R) isotherm models were employed to simulate the equilibrium data. The introductions to the studied isotherm models are presented in Text S2, and the calculated parameters and correlation coefficients (R^2^) are shown in Table 2. For KP, the fitting degrees of the four studied isotherm models followed the sequence: Langmuir > Temkin > Freundlich > D-R model. The Langmuir model described the equilibrium data of KP best among the studied models with R^2^ of 0.9962, indicating the adsorption sites of KP might be homogeneous, and the adsorption process of MG on KP might be dominated by monolayer surface coverage [2]. For NA-KP, the fitting degrees of the four studied isotherm models followed the sequence: Freundlich > Temkin > Langmuir > D-R model. The Freundlich model fitted the equilibrium data of NA-KP best among the studied models with R^2^ of 0.9960, which illustrated that adsorption sites of NA-KP showed different affinities to MG, and multilayer adsorption behaviors might be the dominant mechanism [38,39]. Moreover, the R^2^ values of the Temkin model for both KP and NA-KP were higher than 0.99, which indicated that the adsorption process involved chemisorption and that strong intermolecular forces played an important role during the adsorption process [40].

Moreover, the parameters calculated from the isotherm models could expose more characteristics of the studied adsorption system. As shown in Table 2, the maximum adsorption capacity (q_m_) of NA-KP obtained from Langmuir model was 580.61 mg g^−1^, which was obviously higher than that of KP (297.15 mg g^−1^), and was superior to the agricultural by-product based biosorbents reported in recent literatures (Table S2). Moreover, the separation factors (R_L_) of NA-KP were smaller than that of KP, and both the R_L_ of KP and that of NA-KP were smaller than one. This result suggested the favorability of MG adsorption onto KP and NA-KP, and the adsorption was more favorable for NA-KP [41]. As for the parameters obtained from Freundlich model, both 1/n of KP and NA-KP were smaller than one, which demonstrated the favorability of MG adsorption onto KP and NA-KP [42]. Furthermore, the 1/n of NA-KP (0.17) was smaller than that of KP (0.28), indicating that MG adsorption was more favorable by NA-KP than by KP. Moreover, the q_m_ obtained by D–R model showed that the adsorption capacity of NA-KP was superior than that of KP. In addition, the required sorption energy E was lower in both cases than 8 kJ mol^−1^, which suggested that physical interactions existed during MG adsorption by both KP and NA-KP [43].

### 2.4. Adsorption Thermodynamics

**Figure 4:**
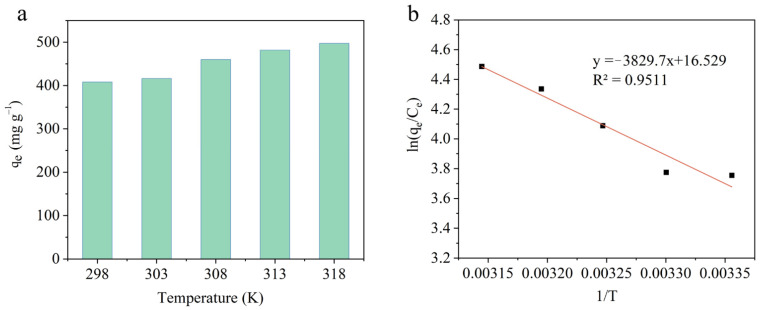
Effect of temperature on adsorption capacities of NA-KP (**a**), and Van ’t Hoff plot (**b**).

Analysis of the effect of temperature and the thermodynamics was performed to further investigate the feasibility of MG adsorption onto NA-KP. As shown in Figure 4a, with the temperature increasing, the adsorption capacities of NA-KP presented a tendency to increase, which illustrates the endothermic nature of the adsorption process. Moreover, the thermodynamic factors, including free energy change (ΔG°), enthalpy change (ΔH°), and entropy change (ΔS°), were calculated by the following Van ’t Hoff equations:

(1) $$K_{c}=\frac{q_{e}}{C_{e}}$$

(2) $$\Delta G{}^{\circ}=-\mathrm{RT}\ln K_{c}$$

(3) $$\ln K_{c}=\frac{\Delta S{}^{\circ}}{R}-\frac{\Delta H{}^{\circ}}{\mathrm{RT}},$$

where K_c_ is the adsorption distribution coefficient (L g^−1^), R is the universal gas constant (8.314 J mol^−1^ K^−1^), and T is the temperature (K) [44].

The linear fitting of lnK_c_ vs. 1/T is shown in Figure 4b, and the calculated thermodynamic parameters of MG adsorption onto NA-KP are presented in Table 3. The negative values of ΔG° at the studied temperature demonstrated the spontaneity and feasibility of MG adsorption onto NA-KP. Moreover, with the temperature increasing, the value of ΔG° became more negative, which indicated that the adsorption process was more spontaneous at higher temperatures. Furthermore, all ΔG° values were in the range of −20 and 0 kJ mol^−1^, which suggested physisorption was important during the adsorption process [26]. The positive value of ΔH° further confirmed the endothermic nature of the adsorption process by NA-KP [45]. Additionally, the positive value of ΔS° suggested the increase of randomness at the interface between solid and solution during the adsorption process [15,46,47].

### 2.5. Selective Adsorption of Cationic Dye by NA-KP

**Figure 5:**
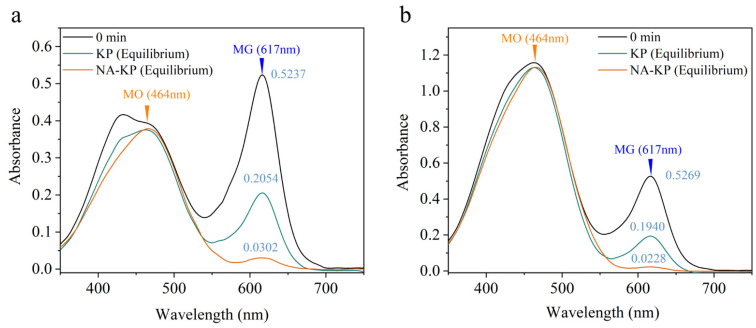
UV-Vis spectra of binary dye mixture before and after adsorption: molar ratio of MG/MO was 3:1 (**a**), and molar ratio of MG/MO was 1:1 (**b**).

Selectivity of adsorbent is critical for the sequential removal of cationic and anionic dyes from wastewater on an industrial scale of separation [48]. In this study, the selectivity of KP and NA-KP for cationic dye (MG) from a mixture of cationic-anionic dye solutions (MG/MO solutions) was investigated and the separation factor was calculated. The UV-Vis spectra of the mixtures before and after adsorption are shown in Figure 5. It can be observed that the concentration of MO was almost unchanged, while the concentration of MG obviously decreased after adsorption, and the selectivity of cationic dye by NA-KP was superior over KP. As shown in Figure 5a, when the molar ratio of MG/MO was 3:1, the separation factors (α_MG/MO_) of KP and NA-KP were 30.03 and 307.13, respectively. Moreover, as presented in Figure 5b, when the molar ratio of MG/MO was 1:1, the separation factors (α_MG/MO_) of KP and NA-KP were 117.79 and 698.10, respectively. Furthermore, the value of α_MG/MO_ by NA-KP was higher when the molar ratio of MG/MO was 1:1 compared to 3:1. This might be due to the comparatively less cationic MG molecules having the advantage of occupying more adsorption sites on NA-KP. Therefore, before achieving the adsorption equilibrium, NA-KP could capture almost all of the MG molecules in a binary dye mixture, and thus facilitate the increase in α_MG/MO_ [49].

### 2.6. Adsorption Mechanism of MG by NA-KP

**Figure 6:**
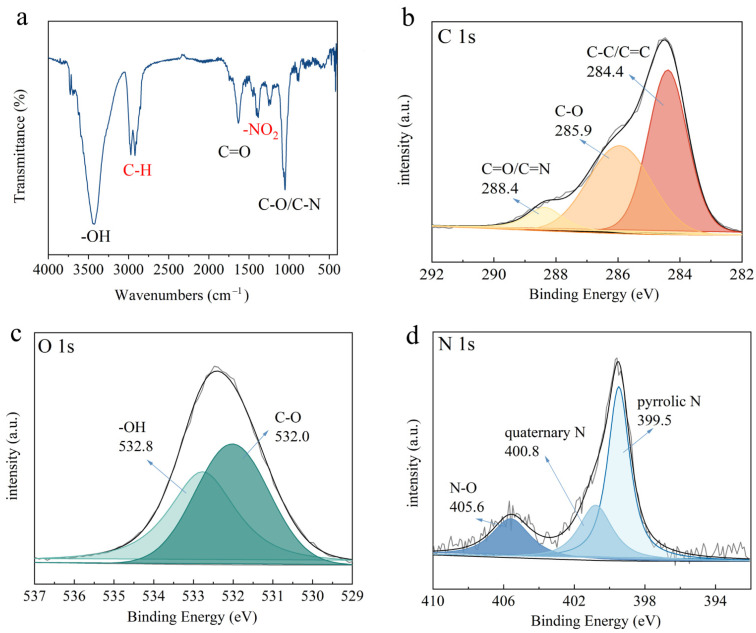
FT-IR spectra of MG@NA-KP (**a**), and XPS spectra of MG@NA-KP: C 1s (**b**), O 1s (**c**), and N 1s (**d**).

In order to gain more insights into the adsorption mechanism of MG by NA-KP, the FT-IR and XPS spectra of NA-KP after MG adsorption (MG@NA-KP) were investigated and analyzed. As shown in Figure 6a, the FT-IR spectrum of MG@NA-KP presented several obvious changes compared to NA-KP. First of all, the -OH peak at 3435 cm^−1^ became sharper and shifted to higher wavenumbers, which suggested that the adsorption process of MG onto NA-KP might involve -OH via hydrogen bonds [8]. Moreover, the peaks observed in the range of 3043–2816 cm^−1^, which were attributed to the vibration of aromatic C-H, became relatively stronger. This might be owing to the structure of the adsorbed MG. Furthermore, the peak at 1385 cm^−1^, observed in the spectra of NA-KP, became obviously weaker in the spectra of MG@NA-KP, which suggested that -NO_2_ might also have played an important role during the adsorption process.

Furthermore, the XPS of MG@NA-KP were evaluated to investigate the surface chemical state changes after adsorption, and the deconvolution spectra of C 1s, O 1s, and N 1s were shown in Figure 6b, Figure 6c, and Figure 6d, respectively. For C 1s, the adsorption of MG changed the binding energies and peak areas, which indicated that there might be π-π interactions between the MG molecules and the benzene rings of NA-KP surface [50]. Moreover, the percentage of peak attributed to C=O/C=N was 8.83% in MG@NA-KP, which was significantly lower than that in NA-KP (18.80%), and shifted to higher binding energy (from 287.8 eV to 288.4 eV). For O 1s, the percentage of peak attributed to -OH was 49.49% in MG@NA-KP, which was also significantly lower than that in NA-KP (62.53%). These results suggested the interactions between MG molecules and oxygen-containing functional groups on the surface of NA-KP [51]. For N 1s, the percentage of peak assigned to N-O became lower after adsorption (from 26.91% to 20.69%). This result confirmed the result of FT-IR analysis and indicated that functional groups containing N might also be involved during the adsorption process.

Considering the results of FT-IR and XPS before and after adsorption, the adsorption process of MG onto NA-KP might include several mechanisms. First, there might be hydrogen bonding between MG molecules and oxygen-containing functional groups (-OH or -COOH) on the surface of NA-KP. Furthermore, π-π interactions might have taken place during the adsorption process. Moreover, the zeta potentials of KP and NA-KP were determined, and the results were −3.42 mV and −52.09 mV, respectively. This result suggested that after HNO_3_ modification, functional groups containing N might contribute electron-withdrawing influence on benzene ring, resulting in a partially negative charge on NA-KP, which was favorable for selective adsorption of cationic dyes through electrostatic interaction [52].

## 3. Experimental

### 3.1. Reagents and Materials

Nitric acid (*w*/*v*, 65%) was purchased from Beijing Chemical Works (Beijing, China), Malachite Green (C_52_H_54_N_4_O_12_, MW = 463.5 g mol^−1^, AR) was obtained from Shanghai Macklin Biochemical Co., Ltd. (Shanghai, China), and Methyl Orange (C_14_H_14_N_3_NaO_3_S, MW = 327.3 g mol^−1^, 96%) was supplied by Aladdin Biochemical Technology Co., Ltd. (Shanghai, China). All reagents were analytical grade and deionized water was used for preparing all solutions.

### 3.2. Preparation of Kiwi-Peel-Based Adsorbents

Kiwis were purchased from a supermarket in Beijing, China. After delivery to the laboratory, the kiwi peel was collected at once and cleaned with deionized water to remove the residual flesh and impurities. The cleaned kiwi peel was dried in an oven at 60 °C, smashed, and filtrated through 80 mesh sieves, then refluxed in methanol for 6 h in a Soxhlet extractor to remove pigments and fats. The obtained particles were dried in an oven at 60 °C for 12 h, which were pristine kiwi-peel-based adsorbents (marked as KP). The modification method was taken from our previous study [13]. A total of 2 g KP was mixed with 200 mL 2 mol/L HNO_3_ and stirred at 25 °C for 2 h, then the temperature was raised to 70 °C and kept there for 2 h. Then, the sample was washed with deionized water several times until the pH value went up to ca. 7. Finally, the nitric acid modified kiwi peel (NA-KP) was dried in an oven at 60 °C for 12 h. Both the prepared KP and NA-KP were stored in an airtight container for further experiments.

### 3.3. Characterization

The morphologies of kiwi-peel-based adsorbents were characterized on an S-4800 scanning electron microscope (Hitachi, Tokyo, Japan). The elemental compositions of kiwi-peel-based adsorbents were analyzed by a FLASH2000 (Thermo, Waltham, MA, USA). Fourier transform infrared spectrums (FT-IR) were determined by a Thermo Nicolet iS10 (Thermo, USA), and the X-ray photoelectron spectrums (XPS) were examined using Scientific K-Alpha (Thermo, USA). Concentrations of MG were determined by I2 UV-Vis spectrophotometer (Hanon, China), and the UV spectrum of the binary mixtures of MG and MO were determined by UV2600 spectrophotometer (Shimadzu, Kyoto, Japan). The maximum adsorption wavelength of MG and MO were 617 nm and 464 nm, respectively.

### 3.4. Batch Adsorption Studies

The adsorption performances of kiwi-peel-based adsorbents were investigated by batch adsorption experiments. The dosages of KP and NA-KP were fixed as 50 mg L^−1^ for kinetic studies, and the volume of dye solution was 500 mL to reduce the impact of sampling volume; for isotherm studies and thermodynamic studies, the volume of dye solution was 200 mL. KP or NA-KP were added into dye solutions and stirred at a speed of 500 rpm. The samples were taken at a preset time or after equilibrium, centrifuged at 5000 rpm for 3 min, and the supernatant solution was taken for concentration determination. The effect of HNO_3_ modification, contact time, initial dye concentrations and temperature were investigated. All experiments were run in triplicate or repeated three times to make sure the data were reliable. The adsorption capacity of the studied adsorbents at time t (q_t_, mg g^−1^) was calculated by Equation (4) [53]:

(4) qt=(C0−Ct)×VM,

where C_0_ and C_t_ (mg L^−1^) are the initial concentrations and the concentration of MG at time t, respectively, V (L) is the volume of the solution, and M (g) is the weight of adsorbent. The equilibrium adsorption capacity of the studied adsorbents (q_e_, mg g^−1^) was calculated by Equation (5) [53]:

(5) qe=(C0−Ce)×VM,

where C_e_ (mg L^−1^) is the equilibrium concentration of MG.

### 3.5. Selectivity Studies

For the selectivity studies, 10 mg KP or NA-KP were added into 200 mL binary mixtures of MG and MO with the molar ratios of MG/MO at 3:1 and 1:1, respectively. The solution was stirred at a speed of 500 rpm, and the UV-Vis spectra of the mixtures before and after adsorption were determined by a UV spectrophotometer. The selective performance of NA-KP for cationic dye is based on separation factor (α) calculated by Equation (6) [48]:

(6) $$\alpha_{\mathrm{MG}/\mathrm{MO}}=\left(\frac{Q_{\mathrm{MG}}}{Q_{\mathrm{MO}}}\right)\left(\frac{C_{\mathrm{MO}}}{C_{\mathrm{MG}}}\right),$$

where Q_i_ and C_i_ (i: MG or MO) are the equilibrium adsorbed dye quantity and equilibrium concentration in the solution, respectively.

## 4. Conclusions

In this study, kiwi peel was used to prepare selective cationic dye adsorbents. Pristine kiwi peel (KP) and nitric acid modified kiwi peel (NA-KP) based adsorbents were characterized and compared, and the results showed that NA-KP possesses more functional groups favorable for adsorption. Adsorption kinetic studies showed the adsorption process of MG onto KP and NA-KP, which followed the pseudo-second-order model and pseudo-first-order model, respectively. Moreover, adsorption isotherm studies suggested that the Langmuir model could describe the equilibrium data of KP best, while the Freundlich model fitted the equilibrium data of NA-KP best among the studied models. Furthermore, the Langmuir maximum adsorption capacity of NA-KP (580.61 mg g^−1^) was superior to that of KP (297.15 mg g^−1^). Thermodynamic studies demonstrated that the adsorption process of MG by NA-KP was feasible, spontaneous, and endothermic. Notably, both KP and NA-KP exhibited selectivity towards cationic dye MG against anionic dye MO, while the selectivity of NA-KP was higher than KP. Additionally, the possible adsorption mechanism of MG onto NA-KP was discussed by comparing the FT-IR and XPS before and after adsorption, which demonstrated that hydrogen bonding, π-π interactions, and electrostatic interaction played important roles during the adsorption process. Consequently, the effective, low-cost, and eco-friendly kiwi-peel-based biosorbent for selective cationic dye removal exhibited numerous potentials in the application of dyeing wastewater treatment.

## Figures and Tables

**Table 1:** Kinetic parameters for MG adsorption on KP and NA-KP.

Kinetic Models	Equation	Parameters	The Value of the Parameters	
			KP	NA-KP
Pseudo-First-Order	$q_{t}=q_{e}\left(1-\exp\left(-k_{1}t\right)\right)$	q_e,cal_ (mg g^−1^)	218.17	332.86
		k_1_ (min^−1^)	0.012	0.010
		R^2^	0.9932	0.9943
Pseudo-Second-Order	$q_{t}=\frac{k_{2}q_{e}{}^{2}t}{1+k_{2}q_{e}t}$	q_e,cal_ (mg g^−1^)	254.81	401.35
		k_2_ (g mg^−1^ min^−1^)	5.86 × 10^−5^	2.78 × 10^−5^
		h (mg g^−1^ min^−1^)	3.80	4.48
		t_1/2_ (min)	66.97	89.63
		R^2^	0.9958	0.9927
Elovich	$q_{t}=\frac{1}{\beta }\ln\left(\alpha \beta t\right)$	α (mg g^−1^ min^−1^)	9.30	9.52
		β (mg g^−1^)	0.020	0.011
		R^2^	0.9791	0.9822

**Table 2:** Isotherm parameters for ENR adsorption onto CSGPB at 298 K.

Isotherm Models	Equation	Parameters	The Value of the Parameters	
			KP	NA-KP
Langmuir	$q_{e}=\frac{q_{m}k_{L}C_{e}}{1+k_{L}C_{e}}$	k_L_ (L mg^−1^)	0.16	0.73
		q_m_ (mg g^−1^)	297.15	580.61
		R_L_	0.08–0.39	0.02–0.07
		R^2^	0.9962	0.9473
Freundlich	qe=kFCe1n	k_F_	92.84	315.62
		1/n	0.28	0.17
		R^2^	0.9773	0.9960
Temkin	$q_{e}=\mathrm{Bln}\left(k_{T}C_{e}\right)$	k_T_ (L mg^−1^)	2.18	50.08
		B	57.56	76.25
		R^2^	0.9904	0.9911
D–R	$q_{e}=q_{m}\exp\left(-{\beta \varepsilon }^{2}\right)$ $\varepsilon =\mathrm{RTln}\left(1+\frac{1}{C_{e}}\right)$	q_m_ (mg g^−1^)	247.55	543.07
		E (kJ mol^−1^)	0.44	1.29
		R^2^	0.9464	0.9032

**Table 3:** Thermodynamic parameters of MG adsorption onto NA-KP.

T (K)	ΔG° (kJ mol^−1^)	ΔH° (kJ mol^−1^)	ΔS° (kJ mol^−1^ K^−1^)
298	−9.30	31.84	0.14
308	−9.51		
318	−10.47		
328	−11.28		
338	−11.86		

## Data Availability

Data is shared in the paper and Supplementary Materials.

## Supplementary Materials

The following supporting information can be downloaded at: https://www.mdpi.com/article/10.3390/molecules28145310/s1, Table S1. The percentages of main elements in KP and NA-KP; Table S2. Comparison of the maximum monolayer adsorption capacities of MG onto various biosorbents [54,55,56,57,58]; Text S1. Details of kinetic models; Text S2. Details of isotherm models.

## Sample Availability

Samples of the compounds are not available from the authors.
