# Supplementary material for: Efficient and Selective Adsorption of Cationic Dye Malachite Green by Kiwi-Peel-Based Biosorbents
Source: Molecules. 2023 Jul 10;28(14):5310. doi: 10.3390/molecules28145310 (PMC10385289; doi:10.3390/molecules28145310)
Supplement: Supplementary file 1 [file molecules-28-05310-s001.zip › molecules-2464187-supplementary.docx]

Supplementary materials

**Efficient and selective adsorption of cationic dye by kiwi peel based biosorbents**

Yanjun Zhao ^a^, Xintong Liu ^b^, Wenhui Li ^a^, Suyun Pei ^a^, Yifan Ren ^a^, Chen Qu ^a^, Chuandong Wu ^a, *^, Jiemin Liu ^a, c, *^

*^a^* *School of Chemistry and Biological Engineering, University of Science and Technology Beijing, No. 30 Xueyuan Road, Haidian District, Beijing 100083, China*

*^b^* *School of Light Industry, Beijing Technology and Business University, No. 33 Fucheng road, Haidian District, Beijing 100048, China*

*^c^* *Beijing Institute of Graphic Communication, No. 1 Xinghua Street (Section 2), Daxing District, Beijing 102600, China*

^*^ Corresponding Author:

wuchuandong@ustb.edu.cn (Chuandong Wu); liujm@ustb.edu.cn (Jiemin Liu).

**Table S1.** The percentages of main elements in KP and NA-KP.

| Adsorbents | C(%) | H(%) | O(%) | N(%) |
| --- | --- | --- | --- | --- |
| KP | 47.83 | 6.32 | 26.22 | 1.02 |
| NA-KP | 48.21 | 6.21 | 26.64 | 2.70 |

**Text S1.** Details of kinetic models

The pseudo-first-order model can be expressed as the following equation:

$$q_{t}=q_{e}\left( 1-exp(-k_{1}t) \right)$$

where *q_e_* (mg g^−1^) and *q_t_* (mg g^−1^) are the adsorption capacities at equilibrium and at time t, respectively; *k_1_* (h^−1^) is the rate constants of pseudo-first-order model.

Meanwhile, the pseudo-second-order model can be expressed as the following equation:

$$q_{t}=\frac{k_{2}{q_{e}}^{2}t}{1+k_{2}q_{e}t}$$

where *q_e_* (mg g^−1^) and *q_t_* (mg g^−1^) represents the same meanings of the pseudo-second-order model, and *k_2_* (g mg^−1^ min^−1^) is the rate constants of pseudo-second-order model.

The Elovich model can be expressed as:

$$q_{t}=\frac{1}{\beta}\ln\left( \alpha\beta\right)+\frac{1}{\beta}lnt$$

where α is the initial sorption rate constant (mg g^−1^ min^−1^) and β is the desorption constant (g mg^−1^).

**Text S2.** Details of isotherm models

Langmuir isotherm model assumes the adsorbent has homogeneous surface with identical adsorption sites and only can be covered by a complete monolayer. The Langmuir isotherm model can be expressed as:

$$q_{e}=\frac{q_{m}k_{L}C_{e}}{1+k_{L}C_{e}}$$

where *C_e_* (mg L^−1^) is the equilibrium concentration of ENR, *q_e_* (mg g^−1^) is the equilibrium adsorption capacity of ENR, *q_m_* (mg g^−1^) is the maximum calculated monolayer adsorption capacity of adsorbent, *k_L_* (L mg^−1^) is the Langmuir constant related to the free energy of adsorption. Besides, the separation factor of Langmuir model (*R_L_*) can reflect the essential characteristics of the Langmuir isotherm. *R_L_* can be obtained by the following equation:

$$R_{L}=\frac{1}{1+k_{L}\cdot C_{i}}$$

where *C_i_* is the initial concentration of ENR (mg L^−1^). The value of R_L_ can indicate the type of isotherm: irreversible (*R_L_* =0), favorable (0< *R_L_* <1), linear (*R_L_* =1), or unfavorable (*R_L_* >1).

In addition, the empirical Freundlich isotherm assumes the adsorbent has heterogeneous adsorbent surface with non-uniform adsorption sites, and describes reversible adsorption process. The Freundlich isotherm can be expressed as:

$$q_{e}={k_{F}C_{e}}^{\frac{1}{n}}$$

where *K_F_* and n are Freundlich constants parameters that related to sorption capacity and sorption intensity, respectively. If 1/*n* < 1, it indicates the adsorption is favorable, and becoming more favorable as its value get closer to zero.

Moreover, the Temkin isotherm model is based on the assumption that the adsorption heat of all molecules in the layer would decrease linearly due to surface coverage. The Temkin isotherm can be expressed as:

$$q_{e}=Bln(k_{T}C_{e})$$

$$B=\frac{RT}{b}$$

where *k_T_* (L mg^−1^) is the equilibrium binding constant related to the maximum binding energy, *B* is the Temkin isotherm constant, R is the universal gas constant (8.314 J mol^−1^ K^−1^), *T* is the temperature (K), and b is the Temkin isotherm constant that related to the adsorption heat.

Finally, the Dubinin-Radushkevich (D-R) isotherm model was also used to fit the experimental data. The D-R isotherm model is widely used to estimate the biosorption mean free energy E and the characteristics of adsorption. The D-R model can be expressed as:

$$q_{e}=q_{m}exp(-\beta\varepsilon^{2})$$

$$\varepsilon=RTln\left( 1+\frac{1}{C_{e}} \right)$$

where β is a constant related to adsorption energy; ε is polanyi sorption potential, which is correlated to the required sorption energy E to move one molecule of solute from infinity to the surface of sorbents. The value of ε and E can be obtained from the following equations:

$$\varepsilon=RT\ln\left( 1+\frac{1}{C_{e}} \right)$$

$$E=\left( 2\beta\right)^{-1/2}$$

where R is the universal gas constant (8.314 J mol^−1^ K^−1^), T is the temperature (K). And the value of E can reflect the whether the adsorption process tends to chemisorption (E > 8 kJ mol^−1^) or physisorption (E < 8 kJ mol^−1^).

**Table S2** Comparison of the maximum monolayer adsorption capacities of MG onto various biosorbents.

| Biosorbent | q_m_  (mg g^−1^) | Temperature  (K) | References |
| --- | --- | --- | --- |
| *Luffa aegyptica* peel (LAP) | 166.67 | 303 | [54] |
| NaOH modified LAP | 161.29 | 303 | [54] |
| Almond peel | 34.60 | 298 | [55] |
| Garlic peel based mesoporous carbon nanospheres | 373.7 | room temperature | [56] |
| Sunflower seed waste derived carbon nanoparticles | 11.07 | 298 | [57] |
| Textile sludge-sawdust chemically produced activated carbon | 530 | 298 | [58] |
| NA-KP | 580.61 | 298 | This work |
